# Editorial: Renal Regulation of Water and Sodium in Health and Disease

**DOI:** 10.3389/fphys.2022.925022

**Published:** 2022-06-08

**Authors:** Sang Heon Suh, Hyun Jun Jung, Weidong Wang, Soo Wan Kim

**Affiliations:** ^1^ Department of Internal Medicine, Chonnam National University Medical School and Chonnam National University Hospital, Gwangju, South Korea; ^2^ Division of Nephrology, Department of Medicine, Johns Hopkins University School of Medicine, Baltimore, MD, United States; ^3^ Institute of Hypertension, Zhongshan School of Medicine, Sun Yat-sen University, Guangzhou, China

**Keywords:** sodium, water, aquaporin, ENaC (epithelial sodium channel), sodium transporter

A fine regulation of water and sodium balance is an integral part of body homeostasis. The kidney plays a pivotal role in the maintenance of water and sodium balance. Various water channels and sodium transporters, such as aquaporins, epithelial Na^+^ channel (ENaC), and sodium-chloride cotransporter (NCC), are differentially expressed and regulated along the renal tubules and collecting ducts, contributing to body fluid homeostasis and blood pressure control (Noda et al., 2010; Warnock et al., 2014; Su et al., 2020; Meor Azlan et al., 2021; Prieto et al., 2021). A robust understanding of the physiology and pathophysiology of renal water and sodium regulation may facilitate the key solutions to unanswered clinical questions in various dysnatremic disorders and hypertension.

In this regard, the Frontier Research Topic, *Renal Regulation of Water and Sodium in Health and Disease*, has been contributed by a total of ten articles, shedding a light to the recent progress in the renal physiology of water and sodium regulation. Frame et al., taking advantage of pharmacologic interventions and physiological measurement in rodent surgical models, addressed the mechanism of natriuresis during acute intravenous sodium infusion, which is renal sympathetic nerves, but not circumventricular organs, and, more specifically, α 1-, but not β-adrenoreceptors. It is intriguing that the role of α 1-adrenoreceptors during acute sodium loading is opposite to the established roles of the renal sympathetic nerves on renal sodium handling *via* renal α 1-adrenoreceptor-evoked sodium reabsorption and renal β 1-adenoreceptors-mediated renin release. Milano et al. found that, in their loss-of-function studies, β 3-adenergic receptor activates NCC in the distal convoluted tubule by promoting phosphorylation *via* its upstream kinases, such as SPAK and WNKs, and that β 1/2-adenergic receptors regulate the expression of NCC, but not the phosphorylation state of NCC. Lu et al. discovered that high phosphate loading activates renin-angiotensin system, and that, by pharmacologic blockade of (pro)renin receptor, this in turn leads to activation of (pro)renin receptor to promotes phosphaturic response *via* stimulation of fibroblast growth factor 23 production and subsequent downregulation of renal Na/Pi-IIa expression. Kristensen et al., *via* various *in vivo* and *ex vivo* experiments, proved that increased expression and phosphorylation of NCC by excess aldosterone is likely an indirect effect of enhanced ENaC-mediated K^+^ secretion and subsequent hypokalemia, concluding the debates on the association between hyperaldosteronism and upregulation of NCC in rodent models.

It is of note that four original articles are featured with collecting duct physiology. Chen et al. demonstrated that, using aquaporin-2 (AQP2) promoter-driven *Cre*-recombinase, mechanistic target of rapamycin (mTOR) from the renal collecting duct principal cell (PC) in mice down-regulates ENaC, and presented the evidence of the mechanisms by which ENaC activity is regulated by mTOR, such as regulation of type 1 serum glucocorticoid regulated kinase, ubiquitination, ENaC channel turnover and apical membrane residency. The selective deletion of mTOR in PC was ultimately sufficient to alter body sodium homeostasis. Soares et al. presented *in vivo* evidence that activation of Gs signaling exclusively in PCs is sufficient to increase ENaC activity and decrease urinary Na^+^ excretion. The expression of Gs-DREADD (designer receptors exclusively activated by designer drugs) protein, which activates intracellular cAMP signal transduction pathway in response to synthetic, but not endogenous, ligand, such as clozapine N-oxide, was specifically induced kidney PCs in *Aqp2-cre* mice. Deen et al. dissected the underlying mechanism of the paradox that water absorption is increased in PCs by prostaglandin E2 (PGE2) in the absence of arginine vasopressin (AVP), but is decreased in the presence of AVP: In the absence of AVP, PGE2 binds to EP4 receptor that is coupled Gs protein, leading to cAMP generation, followed by AQP2 transcription and translocation. In contrast, AVP induces EP1 receptor, which leads to down-regulation of AQP2 expression, and reduces EP4 receptor. In the presence of AVP, PGE2 decreases AQP2 expression by stimulating EP1. Ho et al. defined a common mechanism of AQP2 regulation by α-actinin 4 that bridges short-term (trafficking of the water channel protein aquaporin-2 to the apical plasma membrane of PCs) and long-term (up-regulation of *AQP2* gene expression) response to AVP. This study unveiled that AVP reduces interaction of α-actinin 4 with F-actin, which facilitates F-actin depolymerization and, in turn, apical AQP2 insertion, while freed α-actinin 4 then enters the nuclei where it interacts with glucocorticoid receptor to enhance long-term vasopressin-induced *AQP2* gene expression.

The current Frontier Research Topic also includes two review articles. Tsilosani et al. summarized recent advances in the mechanisms of aldosterone-regulated sodium transport and its relevance with blood pressure, focusing on the signaling pathways involved in aldosterone synthesis and its effects on Na^+^ reabsorption through ENaC. Kim et al. covered the central and nephrogenic mechanisms of drug-induced hyponatremia in drug-induced hyponatremia, emphasizing the importance of the canonical pathway of AQP2 regulation *via* vasopressin V2 receptor in “nephrogenic syndrome of inappropriate antidiuresis.”

Taken together, this Frontier Research Topic expands our knowledge on physiologic function and pathologic adaptation of the renal nephron. The studies collected here are highlighting novel and sophisticated mechanisms of renal water and sodium handling, presenting robust evidence for further investigations.
